# Impact of the expert consensus on polypharmacy and potentially inappropriate medication use in elderly lung cancer outpatients with multimorbidity: An interrupted time series analysis, 2016–2021

**DOI:** 10.3389/fphar.2022.992394

**Published:** 2022-10-05

**Authors:** Fangyuan Tian, Zhaoyan Chen, Rui Tang, Qiyi Feng, Fengbo Wu

**Affiliations:** ^1^ Department of Pharmacy, West China Hospital, Sichuan University, Chengdu, China; ^2^ Department of Epidemiology and Health Statistics, West China School of Public Health and West China Fourth Hospital, Sichuan University, Chengdu, China; ^3^ Precision Medicine Research Center, Sichuan Provincial Key Laboratory of Precision Medicine and National Clinical Research Center for Geriatrics, West China Hospital, Sichuan University, Chengdu, China

**Keywords:** polypharmacy, potentially inappropriate medication, lung cancer, elderly, trend

## Abstract

**Objectives:** Elderly lung cancer patients often have chronic diseases other than lung cancer. Therefore, this kind of population is often accompanied by polypharmacy. This situation and the resulting potentially inappropriate medication (PIM) use are an increasing global concern. In this context, the Chinese Association of Geriatric Research issued an expert consensus on the safety management of polypharmacy. However, the long- and short-term effects of the expert consensus on polypharmacy and PIM use are not clear.

**Methods:** The study was conducted in Chengdu, a city in southwestern China, consisting of prescriptions for elderly lung cancer outpatients with multimorbidity (cancer with other diseases) from January 2016 to December 2021. The 2019 Beers criteria were used to evaluate PIM use, and interrupted time series analysis was used to evaluate the longitudinal effectiveness of expert consensus by measuring the prevalence of polypharmacy and PIM use. We used R software version 4.2.0 for data analysis.

**Results:** A total of 7,238 elderly lung cancer outpatient prescriptions were included in the study. After the publication of the expert consensus, the level (β = -10.273, *P* < 0.001) of the prevalence of polypharmacy decreased, but the trend (β = 0.158, *p* = 0.855) of polypharmacy increased. The prevalence of PIM use decreased abruptly (β = -22.828, *p* < 0.001) after the intervention, but the long-term trend was still upward (β = 0.907, *p* = 0.916).

**Conclusion:** The long-term effects of the publication of the expert consensus on the prevalence of polypharmacy and PIM use in hospitals in Chengdu were not optimal. Future research on interventions rationing polypharmacy and PIM use is needed.

## Introduction

According to epidemiology, although breast cancer has surpassed lung cancer to become the first cancer with new cases worldwide, lung cancer remains the most common cancer in China, with 0.82 million new cases in 2020 (Global Cancer Observatory, 2020; Cao et al., 2021). Cancer is a disease of aging, with the majority of patients age ≥65 years. Cancer incidence is expected to increase by 67% among individuals age ≥65 years from 2010 to 2030 (Hurria et al., 2014). And approximately 20% of lung cancer patients are over 80 years old (Ahmed et al., 2021). This is because with increasing age, elderly individuals are exposed to carcinogens in the environment more, the higher the risk of DNA mutations in the body, and the immunity of the population will decline after entering old age (Decoster and Schallier, 2019). Compared with middle-aged lung cancer patients, elderly patients often have other chronic diseases, so they have to take a variety of medications; this situation is inevitable and very common (Nilsson et al., 2017; Ding et al., 2020). This multidrug combination therapy may increase the probability of drug interactions, some of which will lead to serious consequences. Adverse drug interactions are caused by changes in drug efficacy and adverse reactions caused by drug combinations. Its essence is that the inhibition of drug metabolism leads to adverse reactions or a significant increase in efficacy, or the induction of drug metabolism leads to a relatively insufficient dose, resulting in a significant reduction in efficacy. Liver and kidney dysfunction and body fat changes in elderly patients significantly change drug distribution, metabolism and excretion, increase the risk of drug interactions, and even cause serious clinical consequences such as disability and death (Wildiers et al., 2014; Koczwara et al., 2022).

At present, the definition of polypharmacy is not completely unified, which usually refers to the phenomenon that the same patient uses more than five drugs at the same time (LeBlanc et al., 2015). Although drugs can usually improve the health status of elderly patients, long-term use of polypharmacy may cause a greater burden. Patients with polypharmacy often experience drug-related burdens, including the impact on elderly patients’ quality of life, adverse reactions after taking medication, complex medication programs, and interference with social activities. Therefore, an increasing number of medical and pharmaceutical experts have called on medical staff to pay attention to the risk of polypharmacy and the resulting potentially inappropriate medication (PIM) use in the population, which has brought about a series of drug-related problems (Hyttinen et al., 2016; Muhlack et al., 2017; Wallace et al., 2017) and has put forward management measures to avoid and reduce the damage caused by drug interactions during polypharmacy combination therapy. To meet the needs of clinical practice and further improve the drug safety of elderly patients, an expert consensus on the safety management of polypharmacy for elderly patients was formulated in October 2018. The expert consensus was implemented through the publicity of some medical media at the social level. And In medical institutions, the expert consensus is mainly interpreted by clinical pharmacists to doctors.

At present, there are some studies on the prevalence or trend of polypharmacy and PIM use in Chinese elderly cancer patients (Tian et al., 2022a; Tian et al., 2022b; Tian et al., 2022c). However, the long-term and short-term effects of expert consensus on polypharmacy and PIM use are not clear. The purpose of this study was to explore the trend of polypharmacy and PIM use in tertiary hospitals from 2016 to 2021 to evaluate its impact. Thus, it can provide a basis for further formulating management policies.

## Methods

### Setting and sample

The study was performed to explore the impact of expert consensus on the trends of polypharmacy (which refers to the same patient uses more than five drugs at the same time) and PIM use among elderly (aged ≥65) lung cancer outpatients with multimorbidity (cancer with other diseases) in nine hospitals. These hospitals are tertiary hospitals with complete outpatient departments and electronic information systems in Chengdu. Chengdu is the capital of Sichuan, the largest province in Southwest China, which had a population of 20.93 million and an area of 14,335 square kilometers in 2020. Among them, the population aged ≥65 was 2.85 million, accounting for 13.62%. Compared with 2010, the proportion of people aged ≥65 increased by 3.60 percentage points. Multimorbidity of patients was determined by the numbers of diagnoses in the medical records. All data were retrospectively collected without any possibility of individual identification.

### Data collection and evaluation criteria

This was a descriptive epidemiologic study using cluster random sampling to extract prescription data of elderly lung cancer outpatients from the hospital information system (HIS) from 1 January 2016, to 31 December 2021. And we define polypharmacy by the number of drugs on the prescription. The data were collected by diagnosis type as follows: 1) basic information (region, prescription code, and department source); 2) patient characteristics (age, sex, and diagnosis); and 3) medication characteristics (generic name, trade name, drug specifications, dosage form, administration route, number of prescriptions, prescription expenditure, and frequency of administration). The criteria in the count of prescribed medications as follows: 1) duration of prescription (≤1 month); 2) route of administration (oral medications, injection medications, topical medications and inhaler, etc); 3) medications directly related to treatment for lung cancer were counted as concomitant medications (such as oral tyrosine kinase inhibitor or antiemetic for chemo). 4) Chinese traditional herbal medications were not included. The Chinese Association of Geriatric Research issued an expert consensus in October 2018, providing a reference for further strengthening the management of polypharmacy. The 2019 Beers criteria (American Geriatrics Society Beers Criteria^®^ Update Expert Panel, 2019) were used to evaluate PIM use. The 2019 Beers criteria was selected as it is more suitable for outpatients than other criteria. The prescription in this study were evaluated by potentially inappropriate with PIM use in older adults (table 2 of the 2019 Beers criteria), PIM use in older adults due to drug-disease or drug-syndrome interactions that may exacerbate the disease or syndrome (table 3 of the 2019 Beers criteria), drugs to be used with caution in older adults (table 4 of the 2019 Beers criteria), potentially clinically important drug-drug interactions that should be avoided in older adults (table 5 of the 2019 Beers criteria) of 2019 AGS/Beers Criteria.

### Data analysis

A medication was considered a PIM by one of the PIM classification tables, and it was considered two PIMs by two PIM classification tables, etc … The prevalence of polypharmacy = (number of polypharmacy prescriptions/total prescriptions) ×100%. The prevalence of PIM use = (number of PIM use prescriptions/total prescriptions) ×100%. Interrupted time series (ITS) were used to conduct linear regression analysis on the two time periods before and after the implementation of the expert consensus to analyze the changes and slope changes of polypharmacy and PIM use. The effect of the intervention measures was evaluated by constructing a discontinuous linear regression model, including the level changes before and after the intervention time point and whether the slope of the decline or rise of the event rate or the number of cases with time changed after the implementation of the intervention, to evaluate the impact of the intervention measures on the outcome variables (Zhang et al., 2009). ITS analysis requires that the data used to construct the discontinuous linear regression model meet the requirements of the intervention measures and show a linear trend with time before and after the intervention. When the time series data show a linear trend before and after the intervention, the linear regression model can be used to fit the data to explore the impact of the intervention measures on the outcome variables. In addition, ITS requires that there is no autocorrelation in the sequence. The Durbin-Watson statistic was used to test whether the sequence had first-order autocorrelation. The Durbin-Watson statistic is between 0–4, and its value is close to 2, indicating that there is no autocorrelation. If there was a first-order autocorrelation in the data, generalized least square estimation was used, which was realized by the Prais-Winsten statistic (Hartmann et al., 1980). The intervention time in our study began in January 2019, as the data are counted every quarter, and after the expert consensus was released, it will take some time for medical staff to retrieve and learn. This study covered every quarter continuously for an overall period of 6 years, consisting of 12 quarters before the intervention and 12 quarters after the intervention. We used R version 4.2.0 (R Core Team., 2022) for data analysis, and *p* < 0.05 was considered statistically significant.

## Results

### Basic patient characteristics

A total of 7,238 elderly lung cancer outpatient prescriptions were included in this study. The prevalence of polypharmacy in Chengdu increased from 14.27% in 2016 to 18.04% in 2018 and thereafter decreased to 9.17% in 2021. The prevalence of PIM use increased from 31.94% in 2016 to a peak of 42.67% in 2018 and thereafter decreased to 30.13% in 2021 (Table 1). The percentage of patients by type of lung cancer and the prevalence of metastatic patients in 6 years showed in Table 2.

**TABLE 1:** The prevalence of polypharmacy and PIM use in elderly lung cancer outpatients with multimorbidity.

Time	Polypharmacy		PIM		Total
Year	Quarter	%	n	%	n
2016	1	21.51	37	36.05	62	172
2016	2	13.55	29	37.38	80	214
2016	3	11.15	36	25.7	83	323
2016	4	13.99	41	32.42	95	293
2017	1	14.23	38	37.08	99	267
2017	2	13.21	42	34.28	109	318
2017	3	14.22	30	36.02	76	211
2017	4	20.19	43	36.15	77	213
2018	1	19.49	54	50.54	140	277
2018	2	19.55	69	40.79	144	353
2018	3	14.71	49	38.44	128	333
2018	4	18.59	58	42.31	132	312
2019	1	8.09	28	23.99	83	346
2019	2	7.56	27	23.81	85	357
2019	3	8.36	26	18.33	57	311
2019	4	9.79	42	22.61	97	429
2020	1	8.58	29	24.26	82	338
2020	2	6.23	22	24.36	86	353
2020	3	6.05	19	26.11	82	314
2020	4	8.36	30	23.12	83	359
2021	1	7.87	24	27.87	85	305
2021	2	8.21	22	28.73	77	268
2021	3	9.47	25	31.44	83	264
2021	4	11.04	34	32.47	100	308

PIM, potentially inappropriate medication.

**TABLE 2:** The prevalence of type and metastatic in elderly lung cancer outpatients with multimorbidity.

Type	Year					
	2016(N = 1,002)	2017(N = 1,009)	2018(N = 1,275)	2019(N = 1,443)	2020(N = 1,364)	2021(N = 1,145)
NSCLC and other, n (%)	957 (95.51)	970 (96.13)	1232 (96.63)	1379 (95.56)	1289 (94.50)	1076 (93.97)
SCLC, n (%)	45 (4.49)	39 (3.87)	43 (3.37)	64 (4.44)	75 (5.50)	69 (6.03)
Metastatic patients, n (%)	321 (32.04)	265 (26.26)	344 (26.98)	521 (36.11)	569 (41.72)	433 (37.82)

NSCLC, non-small-cell lung cancer; SCLC, small cell lung cancer.

### The effects of polypharmacy

**FIGURE 1:**
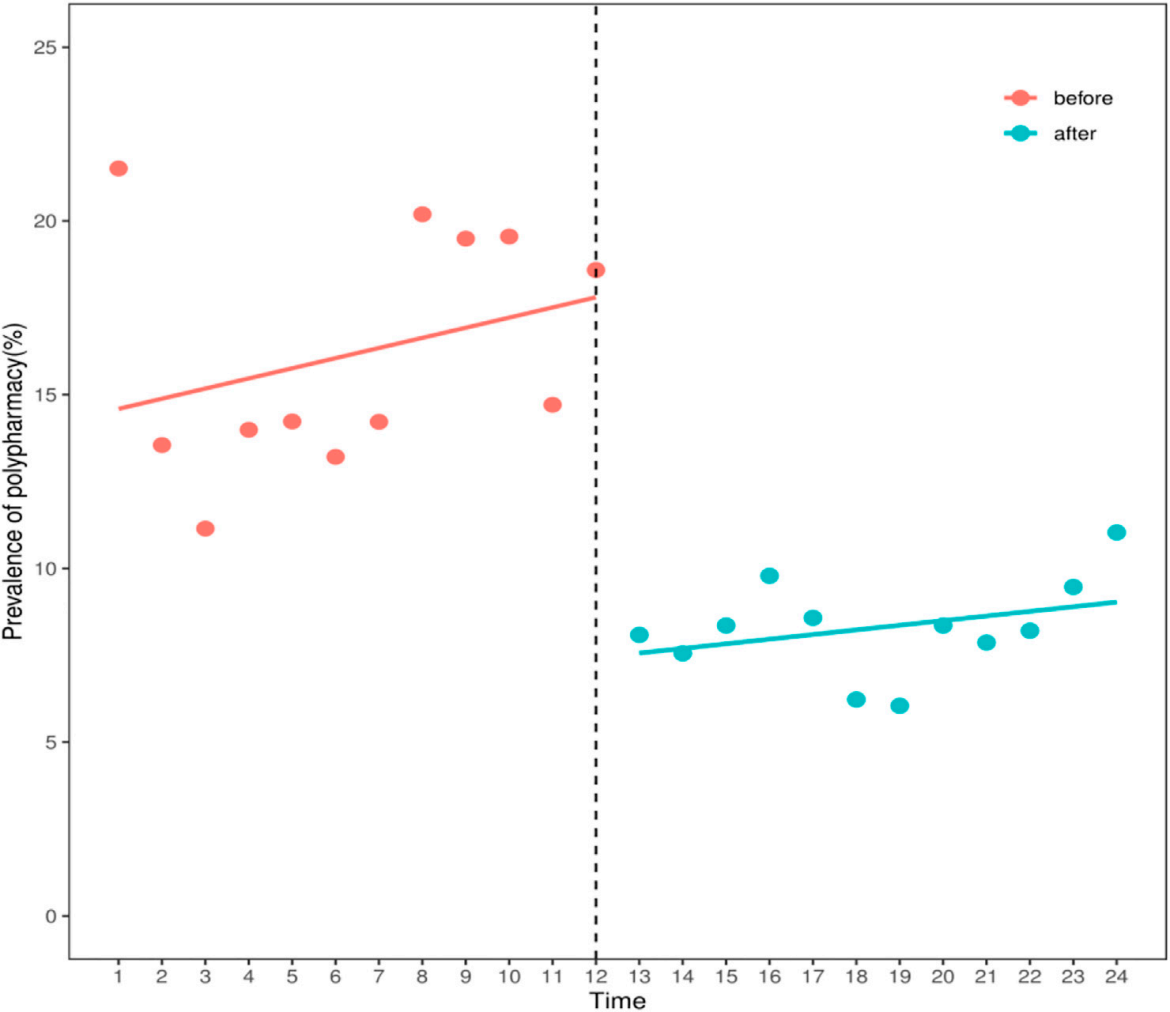
The prevalence of polypharmacy in Chengdu, 2016–2021.

The ITS analysis of the prevalence of polypharmacy of elderly lung cancer outpatients from 2016 to 2021 conducted linear trend judgment and autocorrelation analysis on the data. In the regression analysis of overall polypharmacy prevalence from 2016 to 2021, the Durbin-Watson statistic was 1.304 (*p* < 0.05), suggesting the existence of first-order autocorrelation. Therefore, the Prais-Winsten estimation method was used to correct and construct the interrupted linear regression model. The results showed that before the release of the expert consensus (2016–2018), the slope of the prevalence of polypharmacy was 0.225, showing an upward trend, and the difference was not statistically significant (β1 = 0.225, *p* = 0.377). After the release of the expert consensus, the prevalence of polypharmacy decreased by 10.273% compared with that before the release of the expert consensus, and the difference was statistically significant (β2 = -10.273, *p* < 0.001). After the release of the expert consensus (2019–2021), the slope of the prevalence rate of polypharmacy was 0.225 + (-0.067) = 0.158, showing an upward trend, and the difference was not statistically significant (β3 = -0.067, *p* = 0.855). (Table 3; Figure 1).

**TABLE 3:** Impact of the implementation of the expert consensus on the prevalence of polypharmacy.

Indicators	β	t	*P*
X	14.88	8.049	<0.001
X1	0.225	0.904	0.377
X2	-10.273	-4.355	<0.001
X3	-0.067	0.361	0.855

X1, time variable; X2, intervention measure; X3, slope; β1, the slope before intervention, reflecting the average annual change trend of the prevalence rate before the release of the expert consensus; β2, the amount of level change, reflecting the change in prevalence after the release of expert consensus; β3, the change of slope; (β1+ β3), the slope after the intervention, reflecting the change of the prevalence rate every quarter after the release of the expert consensus.

### The effects of PIM use

**FIGURE 2:**
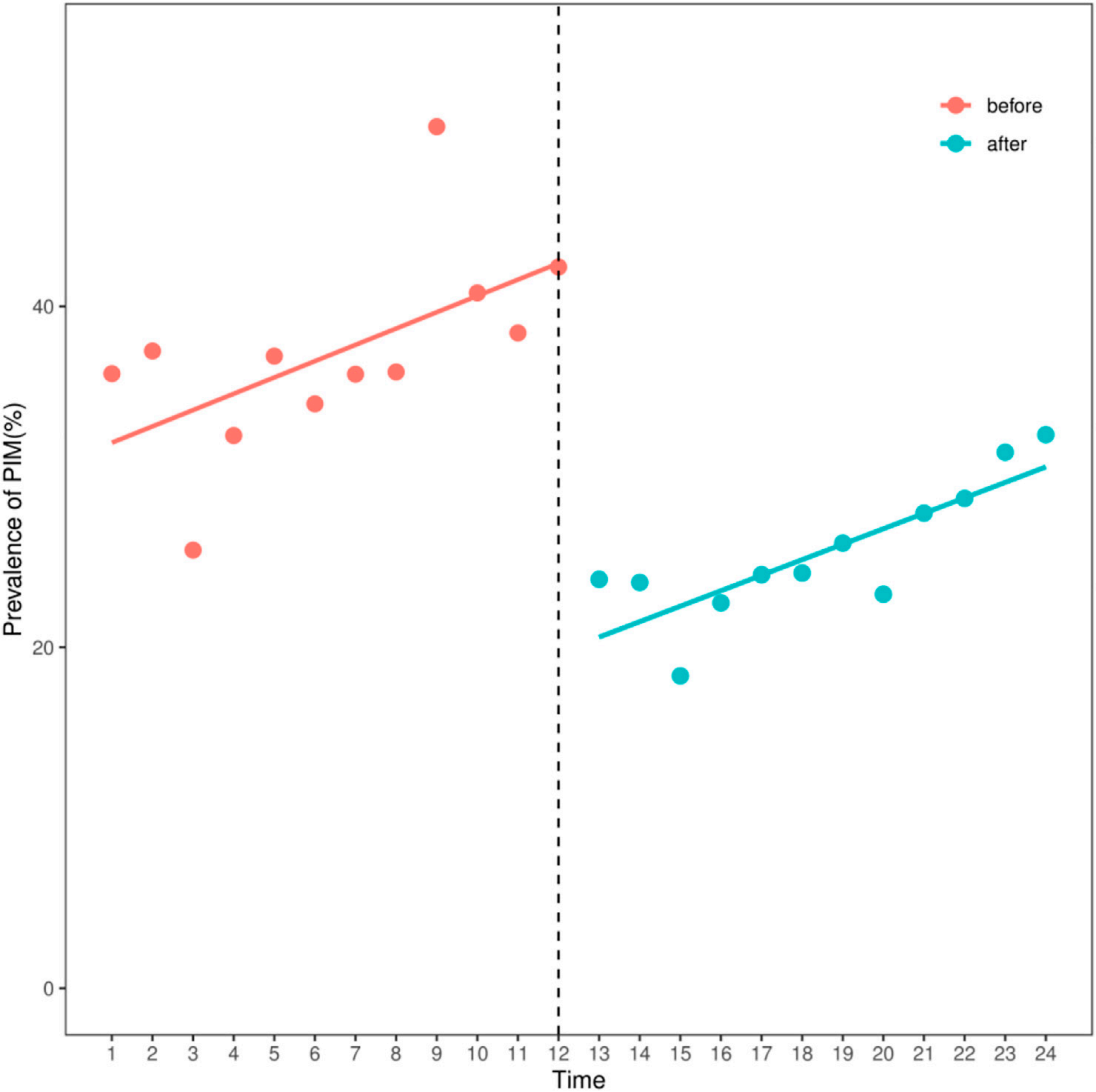
The prevalence of PIM use in Chengdu, 2016–2021.

ITS analysis of the prevalence of PIM use in elderly lung cancer outpatients from 2016 to 2021. In the regression analysis, the Durbin-Watson statistic is 2.052 (*p* = 0.58), indicating that there was no first-order autocorrelation. The results showed that before the release of the expert consensus (2016–2018), the slope of the prevalence of PIM use was 0.957, showing an upward trend, and the difference was statistically significant (β1 = 0.957, *p* = 0.009). After the release of the expert consensus, the prevalence of PIM use decreased by 22.828% compared with that before the release of the expert consensus, and the difference was statistically significant (β2 = -22.828, *p* < 0.001). After the release of the expert consensus (2019–2021), the slope of the prevalence rate of polypharmacy was 0.957 + (-0.05) = 0.907, showing an upward trend, and the difference was not statistically significant (β3 = -0.05, *p* = 0.106). (Table 4; Figure 2).

**TABLE 4:** Impact of the implementation of the expert consensus on the prevalence of PIM use.

Indicators	β	t	*P*
X	31.046	12.791	<0.001
X1	0.957	2.901	0.009
X2	-22.828	-7.053	<0.001
X3	-0.05	-0.106	0.916

X1, time variable; X2: intervention measure; X3, slope; β1, the slope before intervention, reflecting the average annual change trend of the prevalence rate before the release of the expert consensus; β2, the amount of level change, reflecting the change in prevalence after the release of expert consensus; β3, the change of slope; (β1+ β3), the slope after the intervention, reflecting the change of the prevalence rate every quarter after the release of the expert consensus.

## Discussion

To the best of our knowledge, this is the first ITS to assess the impact of expert consensus on polypharmacy and PIM use trends in elderly lung cancer outpatients with multimorbidity. Through a Chinese prescription analysis database, we investigated the impact on the prevalence of polypharmacy and PIM use on the expert consensus of safety management of polypharmacy for elderly patients released in October 2018. After the release of the expert consensus, both the prevalence of polypharmacy and PIM use decreased, especially the prevalence of PIM use. Regarding measures to reduce polypharmacy, studies have shown that unreasonable psychotropic drug polypharmacy in Japan can be reduced by revising the fee of medical services (Hirano and Ii, 2019; Okada and Akazawa, 2022). In addition, there was a study on the application of electronic medicine reconciliation, which reduced the PIM use of elderly inpatients in tertiary hospitals after discharge and transfer to the community in Canada (Welk et al., 2021). Our research results are similar to these studies, proving that the trend of polypharmacy and PIM use can be reduced through some relevant policies or pattern. However, there are some differences in our research. After the release of the expert consensus, polypharmacy and PIM use declined significantly, but with the passage of time, these two increased to a certain extent. Among them, polypharmacy increased slightly, while PIM use increased significantly. First, this expert consensus is about the safety management of polypharmacy, so the impact on polypharmacy is more obvious. Second, polypharmacy can be judged only by the number of drugs prescribed. However, PIM use requires some special judgment criteria, and doctors easily ignore PIM use in the busy work of outpatient clinics. Considering that doctors will not only prescribe drugs for the treatment of lung cancer but also start other related drugs for complications caused by lung cancer, such as pain and depression (Park et al., 2016; Gu et al., 2021; He et al., 2022), it is also difficult to reduce the number of medications.

At present, some studies have shown that the release of expert consensus or some policies can promote the rational application of drugs. One study analyzed the impact of expert consensus on the application trend of carbapenem in medical institutions. Although it promoted the rational use of carbapenems, the long-term effects were relatively general (Ye et al., 2022). This is similar to our research results. Expert consensus has obvious short-term effects on polypharmacy and PIM use, but the long-term effects are poor. Subsequently, the prevalence of both was slowly increasing. However, another study analyzed the impact of national policy on adjusted drug use in tertiary hospitals. The study shows that the use and cost of adjusted drugs have decreased after the introduction of the national management policy (Li et al., 2022). Perhaps this is due to the binding force of the expert consensus being relatively weak compared with the national policy, and the promotion degree is relatively poor compared with the guidelines (Van et al., 2007; Jiang et al., 2015). Therefore, the intervention effect is relatively obvious in the short time after the expert consensus is released, but the long-term effect is relatively poor. Therefore, in view of this situation, when some expert consensus on promoting the rational use of drugs is released, it is best to promote medical personnel in some relevant media to expand the application. In addition, the study and publicity of expert consensus can be increased in clinical use. For example, clinical pharmacists can guide rational drug use through expert consensus to further increase the impression of doctors.

A previous survey on the prevalence of polypharmacy based on the national population showed that there was a certain increase in different countries, increasing from 8.2% in 1999 to 15% in 2012 in the United States (Kantor et al., 2015), increasing from 16.9% in 2006 to 19% in 2014 in Sweden (Zhang et al., 2020), and increasing from 44.9% in 2011 to 47.8% in 2019 in France (Drusch et al., 2021), which is consistent with regional register-based studies on this period in the United Kingdom (a polypharmacy increase from 11.2% in 1995 to 22.8% in 2010; Guthrie et al., 2015) and those using the University Groningen IADB.nl prescription database in the Netherlands (showing an increase from 56.5% in 2012 to 58.2% in 2016; Oktora et al., 2021). This is consistent with the results of our research before the release of the expert consensus, and the prevalence of polypharmacy in elderly lung cancer outpatients with multimorbidity increased from 14.27% in 2016 to 18.04% in 2018. This is mainly because the incidence rate of lung cancer has gradually increased over the years. Meanwhile, the proportion of metastasis patients is relatively high, and a patient with metastatic disease is subject to a much higher number of prescriptions for multiple classes of drugs, not only anticancer but especially related to symptoms and comorbidities, than a patient with local tumor involvement. The treatment of lung cancer includes not only the disease itself but also the complications caused by lung cancer, such as pain and anxiety, which have led to the increase in the prevalence of polypharmacy (Hita-Yañez et al., 2013). Some studies in Europe and the United States have reported a decrease in the prevalence of PIM use (Ble et al., 2012; Hovstadius et al., 2014; Davidoff et al., 2015; Muhlack et al., 2018). With the popularization of the STOPP/START and Beers criteria, clinicians pay more attention to PIM use in elderly patients. However, our research results are different from those of these studies before the release of expert consensus. Our research results show that the prevalence of PIM use in elderly lung cancer outpatients with multimorbidity increased from 31.94% in 2016 to 42.67% in 2018. This is consistent with a study in Ireland that showed that the prevalence of PIM use rose from 32.6% in 1997 to 37.3% in 2012 (Moriarty et al., 2015). As polypharmacy is associated with an increased risk of inappropriate prescriptions, the prevalence of polypharmacy and PIM use in our studies were related, showing an increasing trend. It also shows that expert consensus can play a role in the long-term decline of PIM use.

This study has several limitations that need attention. First, this study only included outpatient prescription data from tertiary hospitals. Therefore, we do not know the impact on polypharmacy and PIM use in community clinics or primary hospitals. Second, we only studied the impact of expert consensus on polypharmacy and PIM use in Chengdu; thus, it may not reflect the impact of expert consensus on the whole country. Third, the data we analyzed were about polypharmacy and PIM use trends in elderly lung cancer outpatients with multimodality, as outpatients rarely have follow-up. It was not clear how this intervention will affect patients in the long term.

## Conclusion

Our study found that before the release of the expert consensus, the prevalence of polypharmacy and PIM use in old lung cancer outpatients with multimodality showed an upward trend. After the release of the expert consensus, these two indicators declined in the short term, but the long-term trend gradually increased. Therefore, to promote the rational use of drugs in elderly lung cancer patients, in addition to issuing relevant expert consensus, some other measures should be taken.(Maleki et al., 2022), (R Core Team, 2022).

## Data Availability

The raw data supporting the conclusions of this article will be made available by the authors, without undue reservation.
